# The King of Causes of Disease

**Published:** 1888-09-08

**Authors:** William Moore

**Affiliations:** K.C.I.E., late Surgeon-General with the Government of Bombay.


### The King of Causes of Disease.

By Sir William Moore, K.C.I.E., late Surgeon-General with the Government of Bombay.

Those two, too dismal authors who observe that "our life is nothing but our death begun," and who liken us all to "shadows hastening to the lower world under the grim convoy of Charon," are no doubt scientifically correct. For, as Ruskin observed, "nothing that lives is, or can be perfect, part of it is decaying, part nascent." But fortunately we are not made quite like the foxglove, which Ruskin instanced as the type of life, because while a third part is in full bloom, a third part is faded, and the remainder bud. Still we are all hastening to that undiscovered country from whose bourne there is no return. Although many smarting under the worries of life would answer the question, "Is life worth living?" in the negative, they mostly do so in a general sense, and even have a strong aversion to that little five-lettered word which expresses the destiny of created beings. Of course, there are insanes who commit suicide, the very fact of the crime having been held as proving aberration of intellect. Yet, as a very general rule, when a person is told by competent authority that his case is a serious one, and if he does not go to Nice he will probably go to heaven—why, he usually journeys to Nice. Prevention is better than cure, and doctors, probably much to their own detriment, are ever endeavouring to obviate the necessity of journeys to Nice, to lessen the pace at which we are hurrying to the undiscovered country, and to render the scientific fact that "while living we are dying," as little apparent as possible. But healthy life depends quite as much on the patient as on the doctor, and especially so as regards avoidance of the "King of Causes of Disease"—Chill. We have heard much recently of bacilli, bacteria, spirilli, micrococci, *cum multis aliis*, of other long-appellated microscopical agencies of disease, "for human pride is skilful to invent most serious names to hide its ignorance," and we may (as bacteriologists would have us believe) or may not, be consumed by an indefinite number of bacteria, or otherwise be poisoned by the exhalations of such bacteria into our blood. There is, however, nothing new under the sun. So long back as 1825, a Dr. Green wrote an essay on "The Nature of Disease," in which, reasoning from analogy of disease caused by visible parasites, he referred numerous other diseases to animalculæ which, with the inferior glasses of the period, he could not see; and he plaintively laments the difficulty of destroying animalculæ, *or vermin of any kind* (the italics are ours), owing to the impracticability of making medicines come in contact with them.

No doubt the atmosphere we breathe is full of solid particles, and probably of germs of different kinds, amongst others of disease also. Voltaire scornfully called the atmosphere a "blue and white mass of exhalations," and if we are to believe all we are told more recently about germs, the wonder is that any are alive to tell or hear. But, fortunately, microbes are in one respect at least like the beings on which they prey. To accomplish their ends they require opportunity, and this is afforded by the vital depression which results from chill. The depression of system caused by the common enemy (chill), gives organisms, if already implanted in the system, the necessary opportunity of establishing themselves; whereas, had there been no chill superadded, the vital force of health would have destroyed the organisms. Whether, however, there be truth or not in the microbe or germ theory of disease, chill *per se* is quite sufficient to excite many maladies. The normal temperature of the human body in health does not vary very much. Unless chilled, its surface is rarely below 98·4 deg. Fah. But as a quarter degree of temperature marks the difference between the fluidity and solidity of water, and as half a grain will turn the balance of a million grains, so a very slight chill of the surface of the body will upset that equilibrium of blood circulation which the continuance of health demands. A chill is nothing more nor less than a minor form of fever. There may be no shivering, no burning after, and no sweating, but a sense of cold water running down the back, alternating with a sense of heat and flushing. Nevertheless, the phenomena of fever are present in a minor degree. Doubtless, the first impression is on the nervous system, but the result is interference with the equilibrium of the circulation, whereby blood is driven from the surface of the body and accumulated unnaturally in internal organs. The effect of local chill is seen in chilblains and frost-bites. If chill of the whole body proceeds beyond a certain extent the termination is not disease, but sleepiness, insensibility, and death. The inherent strength of constitution possessed by many enables them to shake off chills, or, in other words induces reaction, with possibly no more ill effects than a slight cold, or feverishness. Unfortunately, this very strength becomes, by affording long impunity, a source of danger. It renders people neglectful of the means of avoiding chill, and inclines them to brave the strange vicissitudes of our changeable climate, when a reverse procedure would be simply ordinary precaution, and not effeminacy. It is all very well for persons to endeavour to harden themselves, but there is such a thing as overdoing it. There is no necessity for the person who essays to play Othello to black his whole body as well as his face and hands. There may be occasions when exposure must be incurred. Orpheus braved the blazing blasts of Tartarus in quest of his love, and the modern Briton may have equally onerous reasons for braving the freezing blasts of his own climate. But that is no reason why he should not take precautions when he can, which both male and female Britons often neglect doing. The savage is spared many inflictions, such as chimney-pot hats, lawyers' bills, income tax, drains, and clothing. But we are not savages and must pay the penalties of civilisation. Unlike the savage, when we sit in a draught we get a chill; we ride on an omnibus in an east wind and we get a chill; we have wet clothes and a chill follows, for wet clothes are conductors of heat from our body. As a result of chills, or as many express it, "one cold on the top of another," we get fever, bronchitis, pneumonia, rheumatism, sciatica, liver, tetanus, with "mair o' horrible and awfu," as the result of our own imprudence. So do our destinies hang on the merest trifles of daily life, which terminate in chill, until at length, sick unto death, we plaintively wail to the physician, "Canst thou not cleanse the stuff'd bosom of that perilous stuff which bears upon the heart."

Instructions how to avoid chill—the King of Causes of Disease—should surely not be required. Yet, if we watch the conduct of the majority, it would certainly seem to be necessary. Much is often attributed to climate, or, in these latter days, to microbes, which is really the result of want of care, or even, indeed, the absence of ordinary precaution. If people will not for themselves "study well the clime and mould its manners to their obsequious forms," they should, for their own advantage, take the advice which in several books is so plainly put before them. But this is just what the majority, in the pride of a fleeting youth and passing health and strength, will not do. They appear to think "all men mortal but themselves," and they suffer in consequence. Men and women, especially young men and more especially young women, as a rule utterly ignore the most prolific cause of disease—chill, and cannot understand, or at least do not act as if they understood, that a sudden reduction of the temperature of the body is fraught with evil consequences. Although they may have read the classics, in which there are half-a-dozen stories showing there are wishes, the gratification of which is fatal, they do not profit thereby, and if they wish to do anything exposing them to chill they do it. Now one of the principal things rendering one proof against chill is the *mens sana.* The state of the mind is especially connected with affections of the body. It may often be truthfully said of a person, if he had not been dispirited and depressed, he would not have caught cold. As cold is the opportunity of microbes, so depression is the opportunity of chill. The next thing is suitable clothing, of which flannel or woollen is the best. Keeping out of draughts especially when heated, is an obvious precautionary measure. Wet clothes are better conductors of heat from the body than dry clothing, hence the ease with which we get chilled in wet clothing. It is worth recollecting that not only is the atmosphere colder in the early morning hours, but also this is the period when the vitality of the human system is least, hence the desirability of suitable bed clothing, comprising an extra covering which may be drawn up in the early morning. Another thing worth remembering is that hot weather is as dangerous as cold. For during hot weather the skin is in fuller action and more liable to be impressed by slight changes of temperature than in colder seasons. But precautions against chill are not to be measured by line or rule. They are obvious enough to common sense; and the exercise of common sense in the matter would prevent many a ripple in the oceans of humanity, caused by the plunge into the unknown of dear brothers and sisters, the victims of "one cold on the top of another;" additions to the harvest garnered by the King of Causes of Disease.

